# A Possible Role of Crustacean Cardioactive Peptide in Regulating Immune Response in Hepatopancreas of Mud Crab

**DOI:** 10.3389/fimmu.2020.00711

**Published:** 2020-04-30

**Authors:** Yujie Wei, Dongdong Lin, Zhanning Xu, Xiaoman Gao, Chaoshu Zeng, Haihui Ye

**Affiliations:** ^1^College of Ocean and Earth Sciences, Xiamen University, Xiamen, China; ^2^College of Science and Engineering, James Cook University, Townsville, QLD, Australia

**Keywords:** neuropeptide, crustacean cardioactive peptide, hepatopancreas, immunoregulation, arthropod

## Abstract

Crustacean cardioactive peptide (CCAP), a cyclic amidated non-apeptide, is widely found in arthropods. The functions of CCAP have been revealed to include regulation of heart rate, intestinal peristalsis, molting, and osmotic pressure. However, to date, there has not been any report on the possible involvement of CCAP in immunoregulation in crustaceans. In this study, a CCAP precursor (designated as *Sp*-CCAP) was identified in the commercially important mud crab *Scylla paramamosain*, which could be processed into four CCAP-associated peptides and one mature peptide (PFCNAFTGC-NH_2_). Bioinformatics analysis indicated that *Sp*-CCAP was highly conserved in crustaceans. RT-PCR results revealed that *Sp-CCAP* was expressed in nerve tissues and gonads, whereas the *Sp*-CCAP receptor gene (*Sp-CCAPR*) was expressed in 12 tissues of *S. paramamosain*, including hepatopancreas. *In situ* hybridization further showed that an *Sp-CCAPR*-positive signal is mainly localized in the F-cells of hepatopancreas. Moreover, the mRNA expression level of *Sp-CCAPR* in the hepatopancreas was significantly up-regulated after lipopolysaccharide (LPS) or polyriboinosinic polyribocytidylic acid [Poly (I:C)] challenge. Meanwhile, the mRNA expression level of *Sp-CCAPR*, nuclear transcription factor NF-κB homologs (*Sp-Dorsal* and *Sp-Relish*), member of mitogen-activated protein kinase (MAPK) signaling pathway (*Sp-P38*), pro-inflammatory cytokines factor (*Sp-TNFSF* and *Sp-IL16*), and antimicrobial peptide (*Sp-Lysozyme, Sp-ALF, Sp-ALF4*, and *Sp-ALF5*) in the hepatopancreas were all up-regulated after the administration of synthetic *Sp*-CCAP mature peptide both *in vivo* and *in vitro*. The addition of synthetic *Sp*-CCAP mature peptide *in vitro* also led to an increase in nitric oxide (NO) concentration and an improved bacterial clearance ability in the hepatopancreas culture medium. The present study suggested that *Sp*-CCAP signaling system might be involved in the immune responses of *S. paramamosain* by activating immune molecules on the hepatopancreas. Collectively, our findings shed new light on neuroendocrine-immune regulatory system in arthropods and could potentially provide a new strategy for disease prevention and control for mud crab aquaculture.

## Introduction

The neuroendocrine-immune (NEI) regulatory system refers to a complex network formed by the interaction of the nervous system, endocrine system, and immune system (1). The nervous and endocrine systems regulate various physiological processes by releasing neuropeptides, neurotransmitters, and hormones (2). The neuropeptides are usually synthesized and secreted by neurons or neuroendocrine cells and composed of 3–100 amino acid residues (3). As an extracellular chemical messenger, neuropeptides regulate a range of physiological functions, including immunity, growth, reproduction, metabolism, food intake, and circadian rhythm, by activating specific receptors (3). Neuropeptide receptors are mostly G protein-coupled receptors (GPCRs), which constitute the largest family of cell surface receptors. They play a vital role in physiological processes by promoting cellular communication *via* recognizing various ligands, including bioactive peptides, nucleosides, and amines (4).

A large number of studies have shown that neuropeptides also interact with the immune system by binding to receptors of immune cells (5–14). For example, it has been reported that in humans, through binding to their respective receptors, vasoactive intestinal peptide (VIP), pituitary adenylate cyclase-activating polypeptide (PACAP), urocortin 1 (UCN), and adrenomedullin (AM) reduced the production of pro-inflammatory factors (5–7). In rainbow trout *Oncorhynchus mykiss*, prolactin has been shown to increase mRNA expression of *MyD88* and *IL-1*β during *in vitro* infection with the pathogen, *Piscirickettsia salmonis* (8), whereas in Japanese pufferfish *Takifugu rubripes*, neuromedin U elevated the mRNA expression of *IL-6, IL-18*, and *TNF-*α in peripheral blood leukocytes (9). Similarly, in invertebrates, FMRFamide reportedly regulated the expression of immune effectors and apoptosis-related genes *via* P38 mitogen-activated protein kinase (MAPK) signaling pathway in oyster *Crassostrea gigas* (10). In fruit fly *Drosophila*, allatostatin-C receptor 2 (ASTC-R2) played a crucial role in host survival when infected by the pathogenic bacterium *Photorhabdus luminescens* (11). In crustaceans, it has also been reported that crustacean hyperglycemic hormones (CHHs) promoted the elimination of the pathogen *Vibrio harveyi* in the hemolymph and significantly up-regulated the mRNA levels of antimicrobial peptides (AMPs) (PEN4 and crustin) in Pacific white shrimp *Litopenaeus vannamei* (12, 13). For the same species, the silencing of molt-inhibiting hormone (MIH) also led to significant increases in mortality of the shrimp infected by bacterium *Vibrio parahaemolyticus* and white spot syndrome virus (WSSV) (14).

In recent years, more and more evidence suggested that in addition to hemocytes, the hepatopancreas also plays an important role in the immunity of crustaceans (15–21). In addition to function as a digestive gland, the hepatopancreas is also a crucial organ for immunity in crustaceans (16). Indeed, crustacean hepatopancreas is a major source of immune response molecules, including lectins, nitric oxide (NO), stress proteins, antibacterial and antiviral proteins, enzymes, and apoptotic genes (15). Furthermore, many immune-related signal transduction pathways are also found in crustacean hepatopancreas, including MAPK, PPAR, Rap1, PI3K-Akts, cyclic adenosine monophosphate (cAMP), and NF-κB signaling pathway (15, 16, 22). However, more studies are needed to further clarify the immune mechanisms of the hepatopancreas in crustaceans.

Crustacean cardioactive peptide (CCAP) is a cyclic amidated non-apeptide first isolated from the pericardial organs in shore crab *Carcinus maenas* with a function of regulating heartbeat (23). It has since been derived mainly from the nervous system of various arthropods, with confirmed roles of neurohormone and neurotransmitter (24, 25). In recent studies, CCAP mRNA was found in the midgut of cockroach *Periplaneta americana* and Pacific white shrimp *L. vannamei*, as well as in the gills of oriental river prawn *Macrobrachium nipponense* (26–29). CCAP has been shown to be involved in various physiological processes in insects and crustaceans, such as modulation of heartbeat in fruit fly *Drosophila melanogaster* (30) and marine crabs *C. maenas* and *Callinectes sapidus* (23, 31), stimulation of American cockroach *P. americana* midgut contraction and stick insect *Baculum extradentatum* hindgut contraction (26, 32), regulation of ecdysis in prawn *M. nipponense* (29), modulation of oviduct and spermatheca contraction in grasshopper *Locusta migratoria* (33, 34), and increasing survival of shrimp *L. vannamei* subjected to freshwater stress (27).

Like other neuropeptide receptors, CCAP receptor is a GPCR. So far, CCAP receptor has been identified in various insects and crustaceans and has been shown to be involved with its ligand to regulate physiological processes. For instance, knockdown of CCAP receptor reportedly resulted in the loss of CCAP heartbeat regulation function in blood-suck bug *Rhodnius prolixus* (35), and interfering CCAP and its receptor reduced the success rate of ecdysis in red flour beetle *Tribolium castaneum* (36). In mud crab *Scylla paramamosain*, recently, CCAP partial transcript has been found from the cerebral transcriptome database, and its receptor is identified *via* ligand-receptor binding assay by our laboratory (37, 38).

Mud crab *S. paramamosain* is widely distributed in the Indo-Pacific region, and the species is an important mariculture species along the southeast coastal provinces of China (39). Mud crab in aquaculture is vulnerable to diverse bacterial, fungal, and viral pathogens, which could lead to severe economic losses to the industry. In order to prevent and control disease outbreaks in aquaculture, an increasing number of research has focused on the functions and enhancement of the immune system of cultured species (40–42). In this study, we first obtained and characterized the full-length cDNA of *Sp-*CCAP from the cerebral ganglia of *S. paramamosain*. The tissue distribution of *Sp-*CCAP and its receptor (*Sp-*CCAPR) were detected by semi-quantitative RT-PCR, and the locations of *Sp-*CCAPR in the hepatopancreas were further determined by *in situ* hybridization. Subsequently, we investigated *Sp-*CCAPR expression profiles following immune stimulation, and finally the immunomodulatory mechanisms of *Sp-*CCAP and its receptor were evaluated by *in vivo* and *in vitro* experiments. This is the first report on CCAP involvement in immunomodulation in an arthropod, and it potentially provides a new strategy for disease control based on neuroendocrine immunity for mud crab aquaculture.

## Materials and Methods

### Experimental Animals

The animal study protocol has been approved by the Animal Ethics Committee of Xiamen University.

Healthy mud crabs (36.36 ± 2.31 g) at the intermolt stage were purchased from a fish market in Haicang District, Xiamen City, Fujian Province, China. Prior to the experiments, the crabs were acclimated in small tanks (40 × 40 × 60 cm) filled with seawater with salinity 30 ppt and temperature 26 ± 0.5°C for 7 days. During the acclimation period, the crabs were fed fresh field snail *Cipangopaludina chinensis* Gray once daily, and half of tank water was renewed every day.

### Total RNA Extraction and First-Strand cDNA Synthesis

Total RNA from hemocytes and various tissues, that is, eyestalk ganglion, cerebral ganglion, thoracic ganglion, hepatopancreas, gill, stomach, midgut, heart, epidermis, gonad, and muscle, were extracted using TRIzol Reagent (Invitrogen, USA) according to the manufacturer's instructions. The concentrations and quality of RNAs were checked by a Q6000 spectrophotometer (Quawell), and the integrity was assessed by 1.5% (w/v) agarose gel electrophoresis. The first-strand cDNA was synthesized using PrimeScript RT Reagent Kit with gDNA Eraser (TaKaRa) for semi-quantitative RT-PCR and quantitative real-time PCR (qRT-PCR) analyses.

### Cloning the Full-Length cDNA of *Sp-CCAP* and Bioinformatics Analysis

Partial cDNA sequence of *Sp-CCAP* was obtained from the transcriptome database of *Scylla paramamosain* (37). The full-length *Sp-CCAP* cDNA with 1 μg of total RNA extracted from cerebral ganglion was amplified with the SMART™ RACE cDNA Amplification Kit (BD Biosciences). The 3′-race and 5′-race PCR amplification was performed with universal primers [Universal Primer Mix (UPM)] and gene-specific primers for touchdown PCR and nested PCR amplification. PCR products were purified and cloned into the pMD19-T plasmids (TaKaRa). The positive colonies were screened and further confirmed by DNA sequencing. The primer sequences are listed in Table 1.

**Table 1:** Primers for PCRs.

**Name**	**Sequence (5^**′**^-3^**′**^)**
**cDNA cloning**	
3′Out-*Sp-*CCAP	GGCAAGGTTATGGGAGCAACT
3′In*-Sp-*CCAP	GCTCTGTGTATCCAAACATGTGTTG
5′Out*-Sp-*CCAP	TTGCTCCCATAACCTTGCCTC
3′In*-Sp-*CCAP	AACGCAAGGAGGAGGATGGTT
UPM (long)	CTAATACGACTCACTATAGGGCAAGCAGT GGTATCAACGCAGAGT
UPM (short)	CTAATACGACTCACTATAGGGC
**RT-PCR/qRT-PCR**	
*Sp-*CCAP-qF	CGAGGCAAGGTTATGGGAG
*Sp-*CCAP-qR	GATACACAGAGCCACTCAAGAAAT
*Sp-*CCAPR-qF	TCCAAGACTCGCAAATCCA
*Sp-*CCAPR-qR	ATGTCCGTGAGAACACTGAT
*Sp-*β-actin-qF	GAGCGAGAAATCGTTCGTGAC
*Sp-*β-actin-qR	GGAAGGAAGGCTGGAAGAGAG
**qRT-PCR**	
*Sp-*IL16-qF (42)	TGGCAGAGGTTACAGGTCACGGTTAT
*Sp-*IL16-qR (42)	GGAGTCTGGTGTTCGTCACTGTTTCT
*Sp*-TNFSF-qF (43)	CTGTTGTACGTCAGGTCGACTCT
*Sp*-TNFSF-qR (43) *Sp-*LYZ-qF (44)	GGCTCTTCGTATGGGACCTCTG TGCCATCAACCACCACAACT
*Sp-*LYZ-qR (44)	CCCCTTTCCCTTCCACTTCT
*Sp-*ALF1-qF (41)	AACTCATCACGGAGAATAACGC
*Sp*-ALF1-qR (41)	CTTCCTCGTTGTTTTCACCCTC
*Sp-*ALF4-qF (42)	CACTACTGTGTCCTGAGCCGC
*Sp-*ALF4-qR (42)	GTCCTCGCCTTACAATCTTCTG
*Sp-*ALF5-qF (42)	CTTGAAGGGACGAGGTGATGAG
*Sp-*ALF5-qR (42)	TGACCAGCCCATTCGCTACAG
*Sp*-Relish-qF (42)	AGTGGAACAGTGGTCCAGCTG
*Sp*-Relish-qR (42)	CACCACCACTTCACAAATC
*Sp*-Dorsal-qF (42)	TCATCCCCACAATCTGGTGG
*Sp*-Dorsal-qR (42)	TAAGTGCATCTTCCACGTC
*Sp*-P38-qF (45)	TTCACTCCGTCCACCACCTT
*Sp*-P38-qR (45)	GCCCTCGTAACACCTGGTAGAT
***In situ*** **hybridization**	
T7	TAATACGACTCACTATAGGG
SP6	ATTTAGGTGACACTATAG
*Sp-*CCAP-IF	CGACTCCTACTACTTCTAC
*Sp-*CCAP-IR	GATACGGTACTCTTCCAG
**PMD19T**	
RV-M	GAGCGGATAACAATTTCACACA
M13-47	CGCCAGGGTTTTCCCAGTCACG

The ORF Finder (http://www.ncbi.nlm.nih.gov/gorf/orfig.cgi) was used to predict open reading frames (ORFs) and amino acid sequence of *Sp*-CCAP. The amino acid sequence was submitted to predict protein signal peptide with SignalP 5.0 Server (http://www.cbs.dtu.dk/services/SignalP/). The isoelectric point of *Sp*-CCAP was predicted by ExPASy software. The homology amino acid sequences of *Sp*-CCAP from other species in the National Center for Biotechnology Information (NCBI) database were obtained through the BlastX homology search (http://blast.ncbi.nlm.nih.gov/Blast.cgi). These sequences were used to create the multiple sequence alignment by MEGA 7.0 software. Phylogenic trees were constructed *via* the neighbor-joining (NJ) method using MEGA 7.0 software. Bootstrap sampling was reiterated for 1,000 times.

### Tissue Distribution of *Sp*-*CCAP* and *Sp*-*CCAPR* mRNA

Semi-quantitative RT-PCR was used to detect the distribution of *Sp*-*CCAP* and *Sp*-*CCAPR* mRNA in hemocytes and tissues from eyestalk ganglion, cerebral ganglion, thoracic ganglion, hepatopancreas, gill, stomach, midgut, heart, epidermis, gonad, and muscle using *Sp*-*CCAP*-qF, *Sp*-*CCAP*-qR, *Sp*-*CCAPR*-qF, and *Sp*-*CCAPR*-qR as primers (Table 1). The PCR was performed with the Ex-Taq® DNA polymerase (TaKaRa) under the following conditions: pre-denaturation at 94°C for 5 min and 40 cycles consisting of 94°C for 30 s, 58°C for 30 s, and 72°C for 30 s; and the final extension was carried out at 72°C for 10 min. PCR products were resolved on 1.5% agarose gel, and the results were observed and photographed by UV gel imager with water used as a template for negative control and β*-actin* as an internal control. The experiment was repeated three times.

### mRNA *in situ* Hybridization

The 217-bp fragment of *Sp*-*CCAPR* was amplified by PCR and cloned into PGEM-T EASY Vector (Promega) for the subsequent *in situ* hybridization experiment. The riboprobes were synthesized using the DIG RNA Labeling Kit (Roche Diagnostics, Germany) and transcribed by SP6 and T7 polymerases. The hepatopancreas was quickly removed from the crabs and immediately fixed in 4% paraformaldehyde solution for 12 h at 4°C. After being treated with serially diluted ethanol (75, 85, 95, and 100%) and xylene, the sample was embedded in paraffin and sectioned into 0.7-μm continuous sections. Hybridization was subsequently carried out according to the methods reported previously (46) and visualized by the BCIP/NBT Chromogen Kit (Solarbio).

### Immune Challenges With Lipopolysaccharide and Polyriboinosinic Polyribocytidylic Acid Injection

Polyriboinosinic polyribocytidylic acid [Poly (I:C)] (Sigma, USA) was dissolved in crustacean physiological saline (1.13 × 10^−2^ mol/L of KCl, 1.33 × 10^−2^ mol/L of CaCl_2_, 0.44 mol/L of NaCl, 1.0 × 10^−2^ mol/L of Hepes, 2.3 × 10^−2^ mol/L of Na_2_SO_4_, and 2.6 × 10^−2^ mol/L of MgCl_2_, pH 7.4) at 1 mg/ml; and lipopolysaccharide (LPS) (Sigma, USA) was dissolved in crustacean physiological saline at 0.5 mg/ml. Seventy-five crabs were randomly divided into three groups and injected with 100 μl of Poly (I:C), LPS, or crustacean physiological saline (control). In addition, five untreated crabs were used for the initial measurements. Hepatopancreas tissues of five individuals from each treatment group were subsequently randomly sampled at 3, 6, 12, 24, and 48 h for RNA extraction and qRT-PCR analysis. The qPCR used a QuantStudio™ 6 Flex Real-Time PCR (Applied Biosystems) with SYBR® Select Master Mix (TaKaRa). The total reaction volume was 20 μl containing 10 μl of SYBR® Select Master Mix, 2 μl of the five-fold diluted cDNA, 0.5 μl (1.0 × 10^−5^ mol/L) each of the forward and reverse primers, and 7 μl of ultrapure water; and the procedure included 50°C for 2 min; 95°C for 2 min; followed by 40 cycles of 95°C for 15 s, 58°C for 30 s, and 72°C for 30 s; and followed by a melting curve analysis at 60–95°C. A relative transcript level was determined using the 2^−ΔΔCt^ algorithm with β*-actin* from *S. paramamosain* as the internal control. The sequences of the primers used are listed in Table 1.

### Injection of *Sp*-CCAP Mature Peptide

The predicted *Sp*-CCAP mature peptide (PFCNAFTGC-NH2) was synthesized by GL Biochem (Shanghai, China) with a purity of 98% for the subsequent experiments. Forty crabs were randomly divided into two groups: the CCAP treatment group was injected with 100 μl of CCAP mature peptide dissolved in crustacean physiological saline (final concentration in hemolymph about 5 × 10^−6^ mol/L), whereas the control group was injected with crustacean physiological saline of the same volume. Meanwhile, five untreated crabs were randomly selected for the initial measurement. The hepatopancreas tissues of five crabs from each group were sampled at 3, 6, 12, and 24 h after injection for RNA extraction and gene expression analysis (see *Total RNA Extraction and First-Strand cDNA Synthesis* and *Immune Challenges With Lipopolysaccharide and Polyriboinosinic Polyribocytidylic Acid Injection*).

### Hepatopancreas Treated *in vitro* by *Sp*-CCAP Mature Peptide

*S. paramamosain* at the intermolt stage was anesthetized on ice for 10 min, followed by sterilization in 75% ethanol for 5 min. Hepatopancreas tissues were subsequently dissected and washed with crustacean physiological saline before being cut by a pair of scissors into fragments of ~20 mg. The fragments were then precultured at 26°C in a 24-well-plate with 200 μl of L15 medium, which contained penicillin (100 U/ml) and streptomycin (100 μg/ml, Sigma). After an hour, the culture was substituted with L15 medium containing *Sp*-CCAP peptide at one of three concentrations of 10^−6^, 10^−7^, and 10^−8^ mol/L or without adding any peptide (control), which were based on a previous study from our lab (47). Quadruple treatments were used. After 6 h of culture, tissue fragments were collected from each treatment for total RNA extraction and subsequent qRT-PCR analysis, whereas tissue culture medium was also collected for NO concentration measurement. The NO production was determined using the Total Nitric Oxide Assay Kit (Beyotime, China). Briefly, the absorbance of the nitrite was measured with the Griess reaction at OD_540nm_, and the nitrite concentration of each tissue culture medium was then calculated according to the standard curve constructed using NaNO_2_ for the calculation of total NO concentration.

### *In vitro* Antibacterial Assay

The bacterial clearance assay was carried out based on the method described in a previous study (48), with some modifications. That is, after hepatopancreas tissue with *Sp*-CCAP mature peptide was added at three concentrations (10^−8^, 10^−7^, and 10^−6^ mol/L) plus a control without adding *Sp*-CCAP being cultured for 6 h, either *Staphylococcus aureus* or *Vibrio parahaemolyticus* suspension (were stored in our laboratory), both pathogenic to the mud crab, was added to culture wells at the final bacterial concentration of ~3 × 10^4^ cfu/ml per well. After another 3 h of culture, each tissue culture medium was inoculated and cultured on either Luria–Bertani (LB) solid medium (for *S. aureus*) or 2216E solid medium (for *V. parahaemolyticus*) for 12 h at 37°C, and the number of colonies was then observed and recorded. The assay was performed in triplicates for each culture medium.

### Statistical Analysis

All data were presented as mean ± SEM. Statistical differences among treatments were analyzed using one-way ANOVA (followed by Duncan's test) or Student's *t*-test (SPSS 18.0). Differences were considered statistically significant at *p* < 0.05 and highly significant at *p* < 0.01.

## Results

### Molecular Cloning of a cDNA Encoding *Sp-CCAP* Precursor

**Figure 1:**
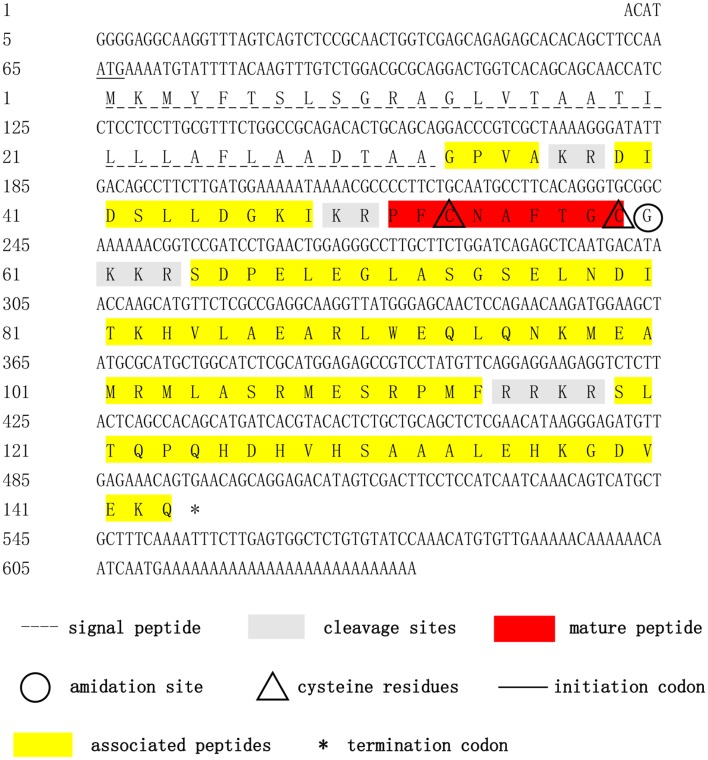
Nucleotide and deduced-amino acid sequences of *Sp-CCAP* cDNA. The initiation codon, termination codon, signal peptide, crustacean cardioactive peptide (CCAP) mature peptide, CCAP-associated peptides, amidation site, cleavage sites, and cysteine residues are marked by different symbols.

The complete cDNA sequence of the *Sp-CCAP* precursor was obtained by using 3′/5′ RACE coupled to nested PCR. The full length of *Sp-CCAP* mRNA is 638 bp with a 64-bp 5′ untranslated region (UTR), a 142-bp 3′UTR, and a 432-bp ORF encoding a protein of 143 amino acids with a calculated molecular weight of 15.84 kDa and a theoretical isoelectric point of 9.43 (GenBank Accession MN923209). The deduced precursor peptide contained a signal peptide of 32 amino acids, four putative dibasic (37KR38 and 49KR50), tribasic (61KKR63), and tetrabasic (115RRKR118) cleavage sites, which could give rise to five peptides, including four precursor-related peptides (CCAP AP1: 33-36; CCAP AP2: 39-48; CCAP AP3: 64-114; and CCAP AP4: 115-143) and one mature peptide containing nine amino acids (PFCNAFTGC-NH_2_) (Figure 1).

### Multiple Alignment and Phylogenetic Tree Analysis

**Figure 2:**
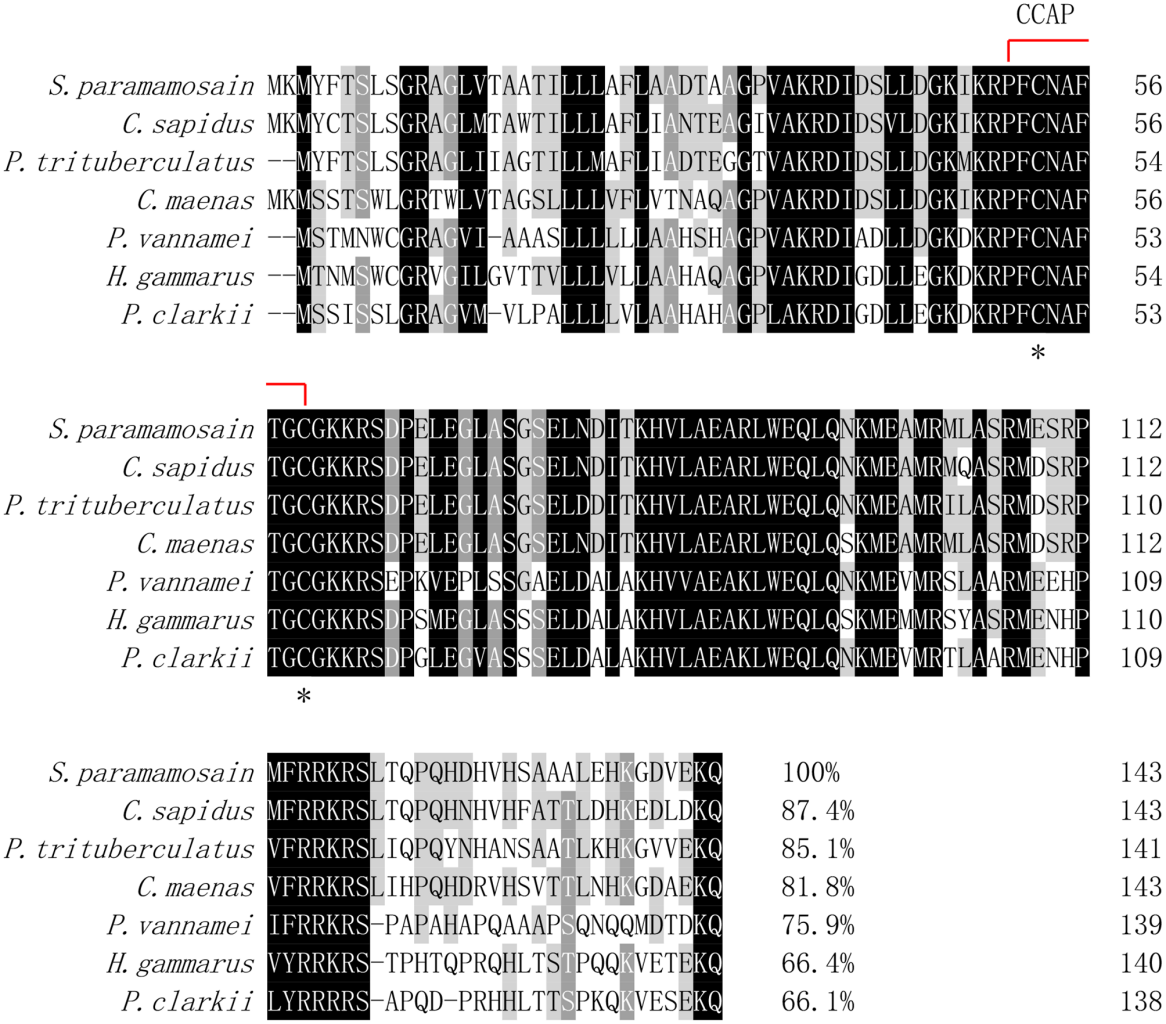
Multiple alignments of the deduced amino acid sequence of crustacean cardioactive peptides (CCAPs) among various crustacean species. The CCAP mature peptide is indicated by the red line; conserved amino acid residues are marked by asterisks. GenBank accession numbers of CCAPs are as follows: *Callinectes sapidus* (ABB46290.1); *Carcinus maenas* (ABB46291.1); *Portunus trituberculatus* (AVK43051.1); *Penaeus vannamei* (ALP06206.1); *Homarus gammarus* (ABB46292.1); and *Procambarus clarkia* (BAF34910.1).

**Figure 3:**
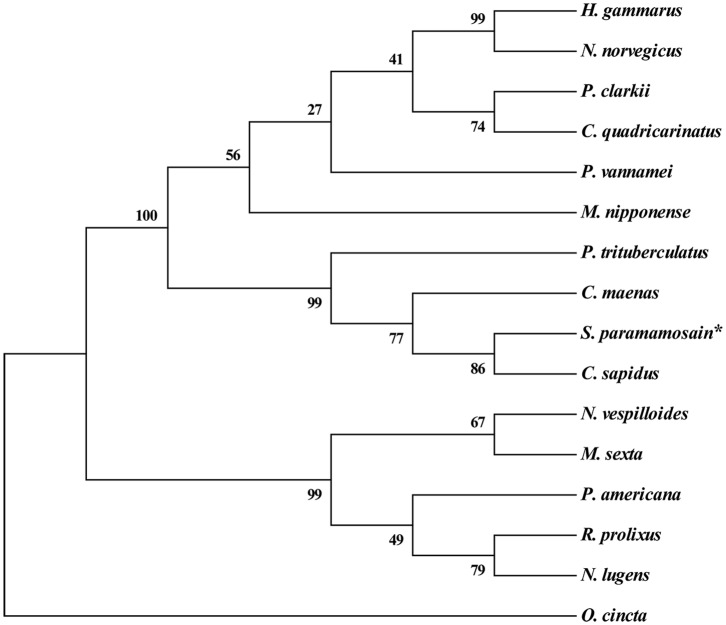
Phylogenetic analysis of crustacean cardioactive peptides (CCAPs) relative to various crustacean and insect species. The sequences used in evolutionary tree analysis include *Callinectes sapidus* (ABB46290.1); *Carcinus maenas* (ABB46291.1); *Portunus trituberculatus* (AVK43051.1); *Homarus gammarus* (ABB46292.1); *Procambarus clarkia* (BAF34910.1); *Nephrops norvegicus* (QBX89037.1); *Cherax quadricarinatus* (AWK57511.1); *Penaeus vannamei* (ALP06206.1); *Macrobrachium nipponense* (ASH96804.1); *Periplaneta Americana* (Q75UG5.1); *Rhodnius prolixus* (ACZ52615.1); *Nilaparvata lugens* (BAO00946.1); *Nicrophorus vespilloides* (XP_017778790.1); and *Orchesella cincta* (ODM98622.1).

Multiple alignment of the amino acid sequences of CCAP precursors from different crustaceans indicated that the CCAP mature peptides were fully identical among the crustacean species used for comparison (Figure 2). Phylogenetic analysis of the amino acid sequences of CCAP precursors among different arthropod species showed that *Sp*-CCAP and other crustacean CCAP clustered into one branch, whereas insect CCAP clustered into another branch (Figure 3).

### Tissue Distribution of *Sp-CCAP*/*Sp-CCAPR*

**Figure 4:**
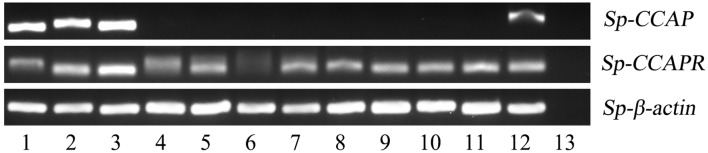
Tissue distribution of *Sp-CCAP* and *Sp-CCAPR* in *S. paramamosain*. 1, eyestalk ganglion; 2, cerebral ganglion; 3, thoracic ganglion; 4, gill; 5, hepatopancreas; 6, hemocytes; 7, stomach; 8, midgut; 9, heart; 10, epidermis; 11, muscle; 12, gonad; and 13, the negative control.

The expression pattern of *Sp-CCAP* among various tissues was determined by semi-quantitative RT-PCR. The results showed that *Sp-CCAP* was expressed in nerve and gonad tissues (Figure 4). To identify the potential target sites of *Sp-CCAP*, the expression pattern of the *Sp*-*CCAPR* transcript was also determined, and *Sp-CCAPR* was found expressed in 12 tissues, including hemocytes and hepatopancreas (Figure 4).

### *In situ* Hybridization of *Sp-CCAPR* in Hepatopancreas

**Figure 5:**
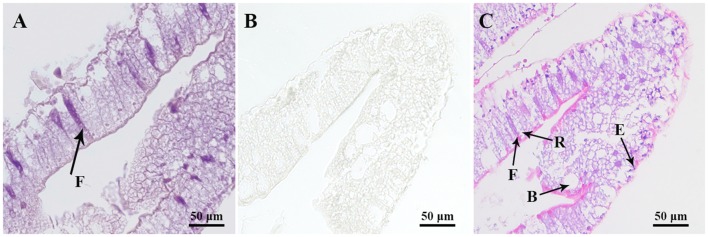
Localization of *Sp-CCAPR* mRNA in the hepatopancreas by *in situ* hybridization. **(A)** Positive signals with the antisense probes. **(B)** Sense probes of *Sp-CCAPR* served as the negative control. **(C)** Histological observation of hepatopancreatic tubule epithelial cells: E, E-cell (E: embryonic); F, F-cell (F: fibrillar); B, B-cell (B: blisterlike); and R-cell (R: resorptive).

To precisely localize the *Sp-CCAPR* transcript in hepatopancreas, *in situ* hybridization was performed. Histological results showed that hepatopancreatic tubule epithelial cells of *Scylla paramamosain* include E-cells (embryonic), F-cells (fibrillar), B-cells (blisterlike), and R-cells (resorptive) (Figure 5C). *In situ* hybridization localized *Sp-CCAPR*-positive signal mainly in the F-cells of hepatopancreas (Figure 5A), whereas in the control group, no such positive signal was found (Figure 5B).

### The Induced mRNA Expression of *Sp-CCAPR* in Response to Lipopolysaccharide and Polyriboinosinic Polyribocytidylic Acid Stimulation

**Figure 6:**
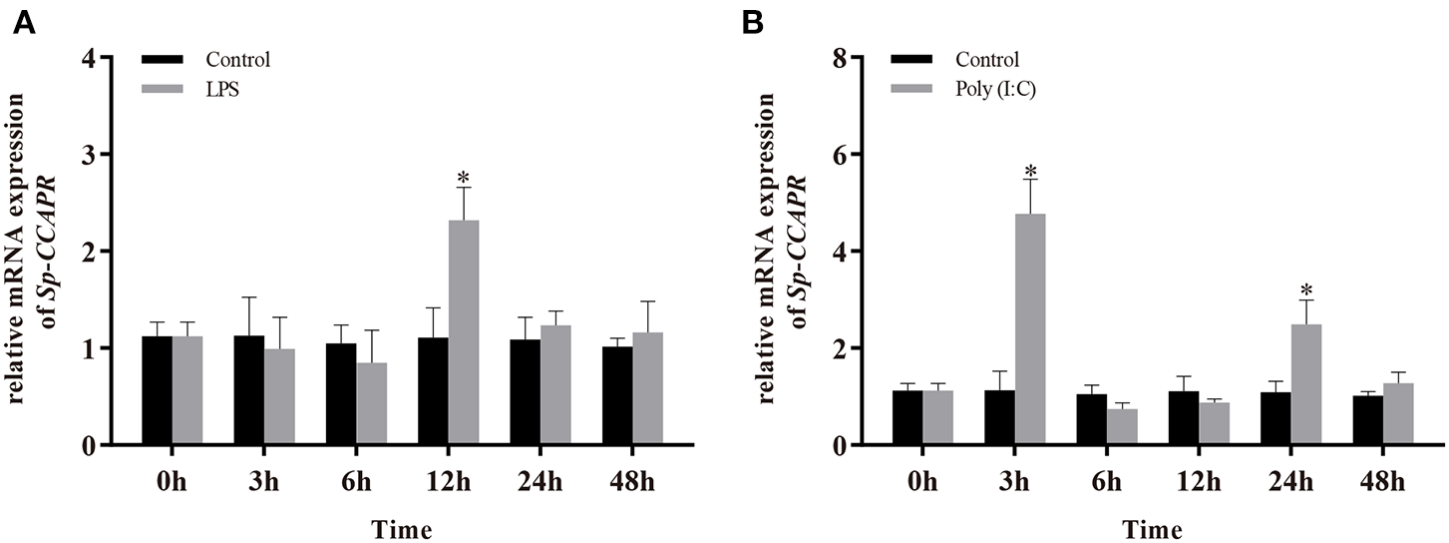
Changes in mRNA expression of *Sp-CCAPR* in the hepatopancreas after lipopolysaccharide (LPS) and polyriboinosinic polyribocytidylic acid [Poly (I:C)] injection. **(A)** After LPS stimulation. **(B)** After Poly (I:C) stimulation. All data are shown as mean ± SEM (*n* = 5); statistical analysis was performed using Student's *t*-test. *indicates significant difference (*p* < 0.05).

Because involvement of neuropeptides in NEI regulation is mainly by binding to their receptors on immune cells, the temporal patterns of *Sp-CCAPR* mRNA expression in the hepatopancreas after LPS and Poly (I: C) injection were investigated. The results showed that following LPS challenge, a significantly up-regulated mRNA expression level of *Sp-CCAPR* was only observed at 12 h, which was 2.09-fold of that in the control (*p* < 0.05) (Figure 6A). However, after Poly (I:C) stimulation, the mRNA expression level of *Sp-CCAPR* was significantly up-regulated at both 3 and 24 h, with 4.16-fold and 2.28-fold increase, respectively, compared with that of the control at the same time point (*p* < 0.05) (Figure 6B).

### The mRNA Expressions of Immune-Related Genes After *Sp*-CCAP Mature Peptide Injection

**Figure 7:**
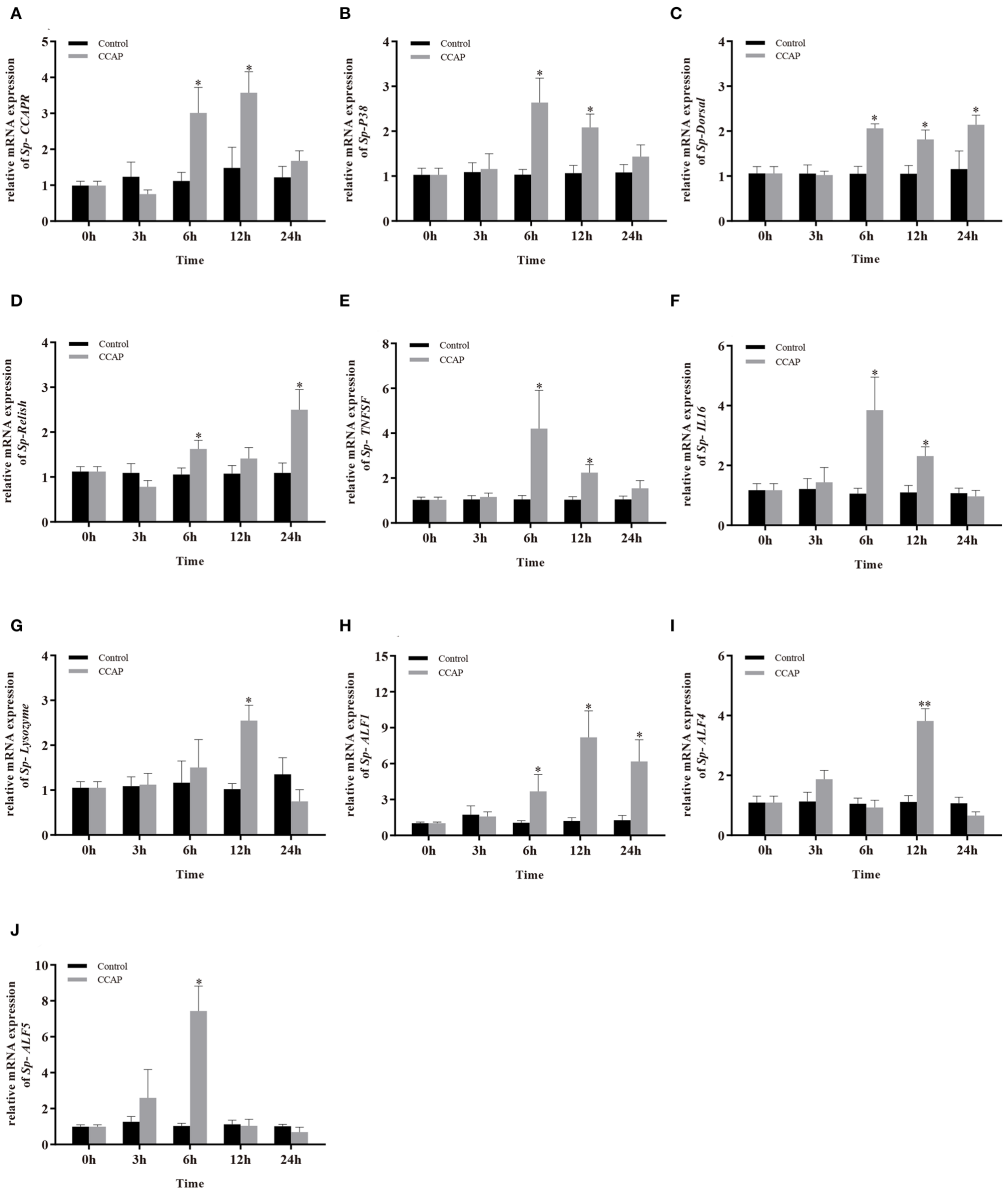
Effects of *Sp-*CCAP injection on the mRNA expressions of immune-related genes in the hepatopancreas. **(A)** *Sp-CCAPR*; **(B)** *Sp-P38*; **(C)** *Sp-Dorsal*; **(D)** *Sp-Relish*; **(E)** *Sp-TNFSF*; **(F)** *Sp-IL16*; **(G)** *Sp-Lysozyme*; **(H)** *Sp-ALF1*; **(I)** *Sp-ALF4*; **(J)** *Sp-ALF5*. All data are shown as mean ± SEM (*n* = 5); statistical analysis by Student's *t*-test. * and **on the top of bars indicate significant (*p* < 0.05) and highly significant differences (*p* < 0.01), respectively.

To evaluate the potential involvement of *Sp*-CCAP in immune regulation, after being injected with synthetic *Sp*-CCAP mature peptide, the changes in mRNA expression levels of immune-related genes, including *Sp-CCAPR*, nuclear transcription factor NF-κB homologs (*Sp-Dorsal* and *Sp-Relish*), member of MAPK signaling pathway (*Sp-P38*), pro-inflammatory cytokines factor (*Sp-TNFSF* and *Sp-IL16*), and AMP (*Sp-Lysozyme, Sp-ALF, Sp-ALF4*, and *Sp-ALF5*), in the hepatopancreas were quantified up to 24 h (Figure 7). The mRNA expression of *Sp-CCAPR* was shown to increase significantly at 6 and 12 h post-*Sp*-CCAP stimulation, which was 2.69-fold and 2.41-fold of that of the control, respectively (*p* < 0.05) (Figure 7A). Similarly, the mRNA expression of *Sp-P38* was significantly up-regulated to 1.45-fold and 1.65-fold of that in the control at 6 and 12 h, respectively (*p* < 0.05) (Figure 7B). Likewise, the nuclear transcription factor *Sp-Dorsal* mRNA expression level was significantly up-regulated at 6, 12, and 24 h (Figure 7C), whereas the *Sp-Relish* mRNA expression level was significantly up-regulated at 6 and 24 h (*p* < 0.05) (Figure 7D). Moreover, pro-inflammatory factor *Sp-TNFSF* and *Sp-IL16* mRNA expression levels were both up-regulated significantly at 6 and 12 h (*p* < 0.05) (Figures 7E,F). Additionally, the mRNA expression level of *Sp-lysozyme* and *Sp-ALF4* increased significantly at 12 h (*p* < 0.05) but returned to normal at 24 h (*p* > 0.05) (Figures 7G,I). The mRNA expression level of *Sp-ALF1* also increased significantly at 6, 12, and 24 h (*p* < 0.05) (Figure 7H). Finally, the *Sp-ALF5* mRNA expression level was sharply up-regulated at 6 h (*p* < 0.05) but dropped back to similar levels to that of the control from 12 h onward (*p* > 0.05) (Figure 7J).

### The mRNA Expressions of Immune-Related Genes and the NO Concentration After *in vitro Sp*-CCAP Mature Peptide Treatment

**Figure 8:**
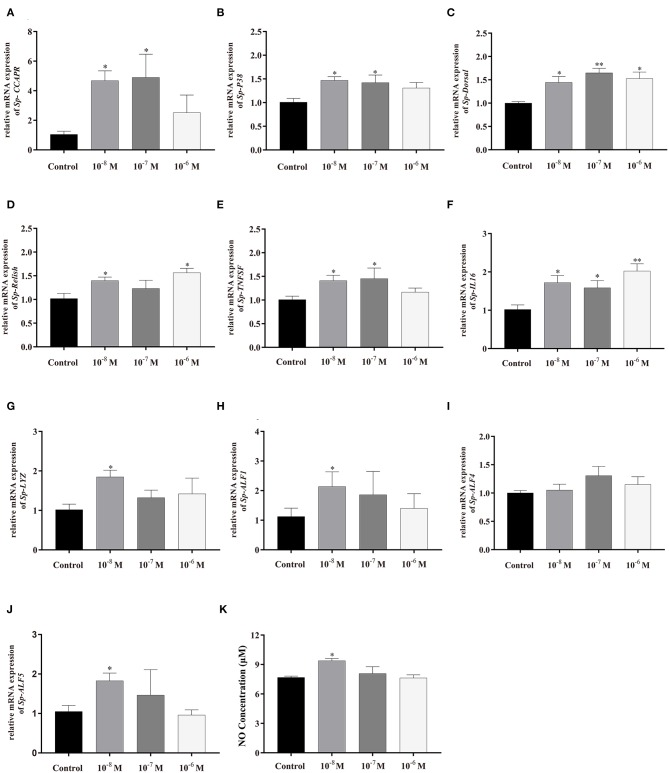
Effects of *Sp-*CCAP addition at different concentrations on mRNA expressions of immune-related genes in *in vitro* cultured hepatopancreas tissues and NO concentration in the culture media. **(A)** *Sp-CCAPR*; **(B)** *Sp-P38*; **(C)** *Sp-Dorsal*; **(D)** *Sp-Relish*; **(E)** *Sp-TNFSF*; **(F)** *Sp-IL16*; **(G)** *Sp-Lysozyme*; **(H)** *Sp-ALF1*; **(I)** *Sp-ALF4*; **(J)** *Sp-ALF5*; and **(K)** the concentration of NO. All data are shown as mean ± SEM (*n* = 4); statistical analysis performed by one-way ANOVA followed by Duncan's test. * and **on top of bars indicate significant (*p* < 0.05) and highly significant differences (*p* < 0.01), respectively.

To further evaluate the immunoregulation function of *Sp*-CCAP, *Sp*-CCAP mature peptide was added to the hepatopancreatic explant cultures at three different concentrations (10^−8^, 10^−7^, and 10^−6^ mol/L), and the expression levels of immune-related genes were measured (Figure 8). The mRNA expression level of both *Sp-CCAPR* and *Sp-P38* was both up-regulated following the addition of *Sp*-CCAP mature peptide at all three concentrations, and significant differences were detected at 10^−8^ and 10^−7^ mol/L as compared with those of the control (*p* < 0.05) (Figures 8A,B). Moreover, the *Sp-Dorsal* mRNA expression level was significantly up-regulated at all three concentrations (*p* < 0.05) (Figure 8C), and the *Sp-Relish* mRNA expression level was significantly up-regulated at 10^−8^ and 10^−6^ mol/L (*p* < 0.05) (Figure 8D). Similarly, the *Sp-TNFSF* mRNA expression level was significantly up-regulated at 10^−8^ and 10^−7^ mol/L (*p* < 0.05) (Figure 8E), whereas the *Sp-IL16* mRNA expression level was significantly up-regulated at all three concentrations (*p* < 0.05) (Figure 8F). Finally, the mRNA expression levels of AMP genes *Sp-lysozyme, Sp-ALF1*, and *Sp-ALF5* were significantly up-regulated at 10^−8^ mol/L (*p* < 0.05) (Figures 8G,H,J), whereas the mRNA expression level of *Sp-ALF4* was not significantly different from that of the control at all concentrations (Figure 8I).

NO is an important gaseous signaling molecule that plays a key role in the innate immune system; NO concentration changes in the hepatopancreas culture media were also determined after adding *Sp*-CCAP mature peptide at different concentrations (Figure 8J). It showed that when treated with *Sp*-CCAP at 10^−8^ M, NO concentration in the medium increased significantly (*p* < 0.05); however, NO content did not significantly vary from the control at the concentrations of 10^−6^ and 10^−7^ mol/L (Figure 8J).

### Clearance of Bacteria Facilitated by *Sp*-CCAP

**Figure 9:**
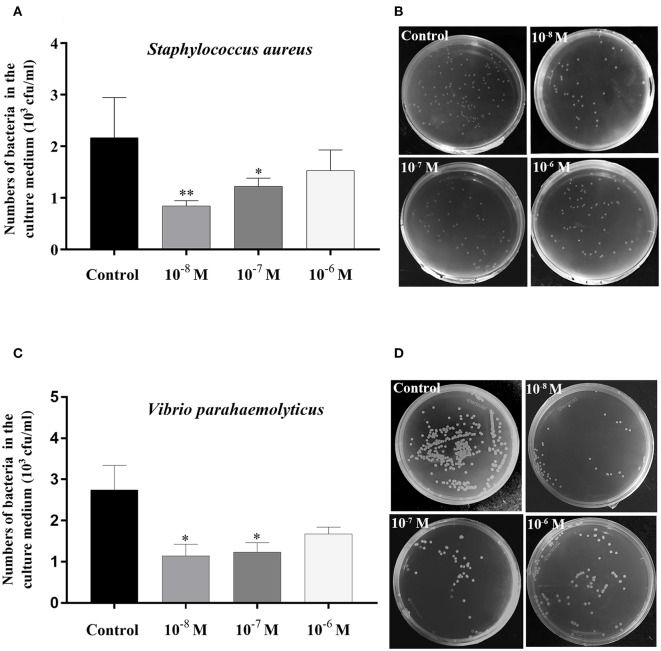
Bacterial clearance capacity of hepatopancreas culture medium treated with different concentrations of *Sp-*CCAP as compared with the no *Sp-*CCAP addition control. **(A)** Results with *Staphylococcus aureus*. **(B)** *S. aureus* colonies grown on Luria–Bertani (LB) plates. **(C)** Results with *Vibrio parahaemolyticus*. **(D)** *S. aureus* colonies grown on 2216E plates. All data are shown as mean ± SEM (*n* = 4); statistical analysis performed by one-way ANOVA followed by Duncan's test. * and **on top of bars indicate significant (*p* < 0.05) and highly significant differences (*p* < 0.01), respectively.

The bacterial clearance capability of each hepatopancreas culture medium with *Sp*-CCAP mature peptide added at 10^−8^, 10^−7^, and 10^−6^ mol/L was evaluated against the control (no *Sp*-CCAP addition) to assess whether up-regulated immune molecules led to enhanced antibacterial capacity. The results showed that based on colony counts, in both cases of *S. aureus* and *Vibrio parahaemolyticus*, bacteria numbers in all tissue culture media with *Sp*-CCAP mature peptide addition decreased compared with those of the control; and the improvement in bacterial clearance capacity was significant when *Sp*-CCAP mature peptide was added at 10^−8^ and 10^−7^ mol/L (*p* < 0.05) (Figures 9A–D).

## Discussion

As a multifunctional peptide hormone, CCAP is known to play an important role in the regulation of various physiological processes in arthropods (23, 26–34). Its immunomodulatory function, however, has never been reported previously; the present study hence appears to be the first to report the involvement of CCAP in regulating hepatopancreas immunity in an arthropod.

In this study, a cDNA encoding CCAP precursor was identified from mud crab *Scylla paramamosain*. In the phylogenetic tree constructed, all CCAP precursor peptides could be divided into two branches of crustacean and insect. The *Sp*-CCAP precursor peptide fell into the branch of crustacean, indicating that *Sp*-CCAP shared high similarity with other crustaceans. By comparing precursor peptides of all sequences from crustaceans, four restriction sites were identified, suggesting that their translation processing modules are highly conserved. CCAP mature peptide sequences (PFCNAFTGC-NH_2_) are identical among different species, which indicates essential roles of CCAP to arthropods.

The distribution of genes in different tissues has a guiding role for exploring the physiological functions of neuropeptides (10). RT-PCR results showed that mRNA transcript of *Sp-*CCAP was mainly expressed in the nervous system and gonads of *S. paramamosain*, whereas the *Sp-*CCAPR mRNA was expressed in a wide range of different tissues. However, in American cockroach *Periplaneta americana* and Pacific white shrimp *L. vannamei*, CCAP mRNA was reportedly expressed in the midgut and the nervous system (26, 27), which suggests that likely there are different physiological regulation pathways of CCAP in arthropod species. In the present study, the detection of mRNA expression of both *Sp-CCAP* and *Sp-*CCAPR in gonads suggested that they may act as an autocrine/paracrine factor to regulate ovarian development, similar to that of short neuropeptide F (sNPF) identified in the same crab species (49). Neurohormones have been found to participate in immune regulation through receptors on hemocytes, the well-known immune-related cells that play crucial roles in host immune defense in crustaceans (50). For instance, in insects, 5-HT receptors were found expressed in the hemocytes, and 5-HT has been shown to modulate hemocyte phagocytosis through 5HT_1B_ and 5-HT_2B_ receptors (51). Similarly, *Es-GPCR89* mRNA was expressed in the hemocytes of Chinese mitten crab *Eriocheir sinensis* and found to mediate cerebral antimicrobial activity (52). In this study, *Sp-*CCAPR was found expressed in the hemocytes, suggesting that *Sp-*CCAP may be involved in the immune regulation of hemocytes as a neurohormone in *S. paramamosain*. In crustaceans, the hepatopancreas is not only the major organ responsible for digestion but also an important immune organ (16). Indeed, in crayfish *Procambarus clarkii*, a putative GPCR gene, HP1R, was found expressed in the hepatopancreas and was suggested to play a role in protecting the host from bacterial invasion (53). In this study, *in situ* hybridization showed that *Sp-*CCAPR mRNA was mainly expressed in F-cells of hepatopancreas, indicating that *Sp-*CCAP may play an immunomodulatory role *via* its receptor on F-cells in *S. paramamosain*.

Increasingly studies have shown that neuropeptides play an important role in the NEI network; typically, they are activated by immune stimuli and bind to their receptors to participate in the innate immune responses (2, 54). In this study, *Sp-*CCAPR was found to distribute in the hepatopancreas of *S. paramamosain*, and its mRNA expression greatly increased after the stimulation of LPS and Poly (I:C), which suggested that both LPS and Poly (I:C) challenges might activate *Sp-*CCAP to bind to its receptor in the hepatopancreas to participate in immune responses. Interestingly, previous studies have reported similar results in other species. For example, HPR1 gene in the hepatopancreas of *P. clarkii* was significantly up-regulated by stimulation with *Aeromonas hydrophila* (53), the mRNA of *LPSenhR-1* was significantly up-regulated after LPS stimulation in rainbow trout *Oncorhynchus mykiss* (55), and Poly (I:C) stimulation induced significantly higher neurokinin-2 receptor mRNA expression in human dendritic cell (56). It is well-known that the neuroendocrine system regulates the immune responses by releasing neuropeptide hormones, whereas the immune system activates the neuroendocrine system by secreting cytokines, thus forming a circular network of neuroendocrine and immune regulation (57). *Sp-*CCAPR showed two expression peaks at 3 and 24 h after Poly (I:C) stimulation; it is speculated that *Sp-*CCAP/*Sp-*CCAPR can respond quickly to Poly (I:C) stimulation and be indirectly affected by Poly (I:C)-induced immune factor activation.

In this study, the immunomodulatory effect of *Sp*-CCAP was further studied by *in vivo* injection and *in vitro* culture experiment. In the *in vivo* experiment, *Sp*-CCAP was found to significantly induce the expression of its receptor *Sp-CCAPR*, P38 MAPKs (*Sp-P38*), and nuclear transcription factor NF-κBs (*Sp-Dorsal* and *Sp-Relish*) in the hepatopancreas, which suggested that by stimulating the expression of *Sp-*CCAPR, *Sp-*CCAP likely induced increased amount of *Sp-*CCAPR on the cell membrane in the hepatopancreas, thus greatly enhancing the activity of signaling pathway mediated by *Sp-*CCAP. On the other hand, P38 MAPKs as a member of MAPK superfamily, can associate extracellular signals with intracellular mechanisms (58) and play a crucial role in the inflammatory response and the host defense against pathogen infections (59). The NF-κB pathway is an essential pathway for the innate immune response to pathogen invasion in both vertebrates and invertebrates (60). Therefore, in order to explore the possibility that immune molecules regulated by *Sp-*CCAP and its receptor *Sp-*CCAPR signaling pathway, the mRNA expression levels of several immune molecules with pro-inflammatory and antibacterial properties, that is, *Sp-IL16, Sp-TNFSF*, and AMPs, were detected. Of these immune molecules, AMPs are key effector molecules that induce innate immunity in various invertebrates (61). As one of the AMP family, the anti-LPS factor (ALF) is well-known to possess a wide range of antibacterial, antifungal, and antiviral properties (62). Another AMP, lysozyme, reportedly protects organisms by destroying the cell walls of infectious bacterial pathogens (63). Meanwhile, interleukin-16 (IL-16), a pleiotropic cytokine, plays essential roles in the regulation of various innate immune processes (64), and has been reported to play a strong positive role in antibacterial responses in *L. vannamei* (65). Relish and Dorsal are invertebrate NF-κB homologs, they function as essential transcription factors on mediating the activation of AMP genes in crustaceans (66), and the expression of IL-16 is dependent on the NF-κB pathway (67). The P38 MAPK plays a key role in a variety of immune responses by regulating the production of pro-inflammatory cytokines, including TNFs and IFNs (58, 59). In this study, the up-regulation of nuclear transcription factor NF-κBs, pro-inflammatory factor IL-16, and AMP genes suggests that *Sp-*CCAP might influence the expressions of *Sp-IL16* and AMPs by mediating the NF-κB signaling pathway, whereas the up-regulation of *Sp-P38* and *Sp-TNFSF* suggests that *Sp*-CCAP might affect the expression of *Sp-TNFSF* by mediating the P38 MAPK signaling pathway. Together, these results suggest that *Sp-*CCAP possibly activates and induces inflammatory and antimicrobial responses in *S. paramamosain*. Interestingly, the *Sp-*CCAPR expression level was not significantly elevated at 24 h post *Sp*-CCAP mature peptide injection, whereas *Sp-Dorsal*, S*p-Relish*, and *Sp-ALF1* expression levels were still up-regulated. This phenomenon may be explained by the possible accumulation of sufficient receptor proteins on the cell membrane, thus promoting these gene expressions.

The results of the *in vitro* experiment were similar to those of *in vivo* experiment; that is, adding *Sp*-CCAP mature peptide to the hepatopancreas cultures could promote the expressions of signal pathway-related genes and immune effector molecules. They provided further evidence that *Sp*-CCAP was involved in hepatopancreas immunity of *S. paramamosain*. Moreover, NO is an important gaseous signal molecule that plays anti-bacterial and inflammatory roles in invertebrates (68). In the present study, it was found that NO concentration in the hepatopancreas culture medium increased significantly when *Sp*-CCAP mature peptide was added at 10^−8^ mol/L. It provided additional evidence from the point of view of gaseous signal molecules that *Sp*-CCAP likely plays a significant role in the hepatopancreas immunity of *S. paramamosain*.

Finally, the *in vitro* bacterial clearance experiment showed that the up-regulation of immune effector molecules in the hepatopancreas mediated by *Sp*-CCAP signaling pathway could effectively resist bacterial infection. Indeed, the results demonstrated for the first time that neuropeptides play antibacterial roles in the hepatopancreas of a crustacean, likely *via* regulating immune-effector molecules.

In invertebrates, on the one hand, when neuropeptides act on immune cells, neuropeptide receptors on the membrane can activate the G-protein Gα_s_/Gα_i_ subunit and react with adenylate cyclase to increase or decrease intracellular cAMP concentration (69). On the other hand, when neuropeptides bind to Go/Gq protein-coupled receptors on immune cells, they can activate the activity of phospholipase C, thereby altering intracellular Ca^2+^ concentration (69). The changes of these secondary messengers (cAMP and Ca^2+^) activate a series of signaling pathways, such as MAPK (JNK, ERK, and P38) and NF-κB signaling pathway, through cascade amplification. They synergistically promote the release of inflammatory factors and immune factors (70). CCAPR was first identified in *Drosophila* but has since been identified in many other insects; it is known that the involvement of CCAPR in various physiological processes, such as molt and heartbeat regulation, is mediated by CCAP (35, 36, 71). A previous study has shown that the binding of *Sp*-CCAP to *Sp*-CCAPR activated the cAMP level and Ca^2+^ signal response in the cytoplasm of *S. paramamosain* (38). Therefore, in this study, activating P38 MAPKs and NF-κB signaling pathways by the binding of *Sp*-CCAP to *Sp*-CCAPR were likely *via* cAMP and Ca^2+^ concentration changes in hepatopancreas cells, which promoted the expression of *Sp-IL16, Sp-TNFSF, Sp-LYZ, Sp-ALF1, Sp-ALF4*, and *Sp-ALF5*, hence enhancing the immune responses of *S. paramamosain* to pathogen infection.

In summary, the present study provides the first evidence that CCAP plays an immunomodulatory role in the hepatopancreas in a crustacean. It suggests that CCAP might activate immune effector molecules mediated by the P38 MAPK pathway and NF-κB pathway in the hepatopancreas to resist pathogen infection. This study also potentially provides a new strategy for disease control from the perspective of neuroendocrine immunity for the mud crab aquaculture.

## Data Availability Statement

The datasets generated for this study can be found in the National Center for Biotechnology Information GenBank Accession MN923209.

## Ethics Statement

All the animals used in this study have been approved by the Animal Ethics Committee of Xiamen University.

## Author Contributions

YW and HY designed the experiments. YW and DL performed the experiments. YW and ZX analyzed the data. XG contributed reagents/materials tools. YW and HY contributed to the discussion. YW wrote the manuscript. HY and CZ revised the manuscript. All the authors read and approved the final manuscript.

## Conflict of Interest

The authors declare that the research was conducted in the absence of any commercial or financial relationships that could be construed as a potential conflict of interest.
